# A multi-sensory dataset for the activities of daily living

**DOI:** 10.1016/j.dib.2020.106122

**Published:** 2020-08-02

**Authors:** Marco Ruzzon, Alessandro Carfì, Takahiro Ishikawa, Fulvio Mastrogiovanni, Toshiyuki Murakami

**Affiliations:** a Department of Informatics, Bioengineering, Robotics and Systems Engineering, University of Genoa, Via Opera Pia 13, 16145 Genoa, Italy; b Department of Electronics and Electrical Engineering, Keio University, 3-14-1 Hiyoshi, Kohokuku, Yokohama, Kanagawa 223-8522, Japan

**Keywords:** Activities of daily living, Inertial measurement unit, Wearable sensing, Human activity recognition, Human locomotion, Accelerometer, Smart home

## Abstract

The article describes a multi-sensory dataset related to the Activities of Daily Living (ADL). These are the activities that contribute to an assessment of the overall status of elderly or people with special needs, possibly suffering from mild cognitive impairments. Typical basic ADLs include walking, such postural transitions as getting up or sitting down, as well as behaviours related to feeding, such as drinking or eating with knife and fork, or personal hygiene, e.g., teeth brushing. The collection process adopted for building this dataset considers nine ADL-related activities, which have been performed in different locations and involving the usage of both left and right arms. The dataset acquisition involved 10 volunteers performing 186 ADL instances, for a grand total of over 1860 examples. The dataset contains data from six 9-axis Inertial Measurement Units (IMUs), worn by each volunteer (two for each arm, one on the back and one on the right thigh). The dataset features an accurate data labelling done via manual annotation performed thanks to videos recorded by an RGB camera. The videos recorded during the experiments have been used only for labelling purposes, and they are not published.

**Specifications Table**

**Table:** 

Subject	Engineering.
Specific subject area	Human-Computer Interaction, Activity Recognition, Ambient Intelligence.
Type of data	Table.
How data were acquired	Six 9-axis IMUs (TSND 151); RGB Camera (Logitech HD Pro Webcam C920).
Data format	Raw and processed.
Parameters for data collection	The data collection process considered 9 ADL (related to walk, sit down, stand up, open a door, close a door, pour water, drink using a glass, teeth brushing and clean a table). The order in which the activities are performed has been chosen to ensure a high variety, and volunteers have been asked to perform the activities with both their hands independently of their dominant hand.
Description of data collection	The dataset has been collected with 10 healthy volunteers. Each volunteer performed an overall 186 ADL-related instances of activity while wearing 6 IMU sensors (two on each arm, one on the back and one on the right thigh). The experiments have been recorded with an RGB camera used for data labelling.
Data source location	Department of Electronics and Electrical Engineering, Keio University, Hiyoshi, Kohokuku, Yokohama, Kanagawa, Japan (35.555659, 139.653391).
Data accessibility	Repository name: Mendeley Data Data identification number: 10.17632/wjpbtgdyzm.1 Direct URL to data: https://data.mendeley.com/datasets/wjpbtgdyzm/1

## Value of the data

• The multi-sensory dataset allows for grounding ADL-related studies, including the development of data-driven techniques for the recognition of ADL in assistive scenarios.
The multi-sensory dataset can be used by researchers in machine learning as a benchmark for ADL recognition, and by physicians to use motion models of heathy subjects as a benchmark for motion analysis.
The high number of considered ADL classes, the number of subjects, considering ADL execution with both arms and the different order followed by the subjects to execute the various actions guarantee a high variety of the dataset.

## 1 Data description

**Fig. 1:**
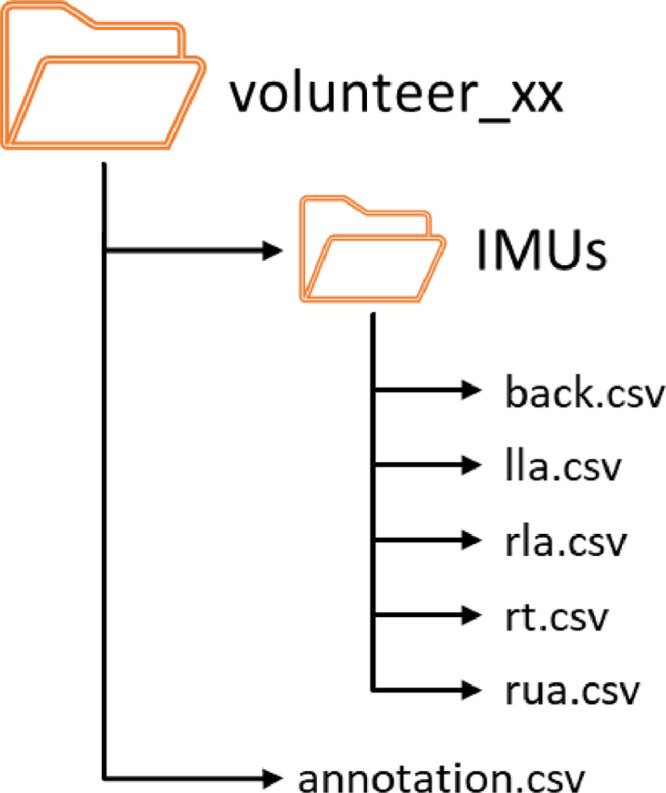
Dataset structure for a generic volunteer.

In this article, we present a multi-sensory dataset concerning the execution of actions related to the Activities of Daily Living (ADL) [1]. The dataset contains multiple instances of the 9 ADL-related actions presented in Table 1 and performed by 10 volunteers. Each volunteer performed each activity at least 14 times, with the notable exception of the *Walk* activity that has been performed 40 times, in different sequences and alternating the hand wearing the IMUs. The dataset published on MendeleyData^1^ is organized as described in Fig. 1, and for each volunteer the dataset contains 7 comma-separated value (csv) files, i.e., one file for each of the 6 IMU sensors worn by the volunteer on different body parts, as described in Fig. 1, namely left lower arm (lla.csv), left upper arm (lua.csv), right lower arm (rla.csv), right upper arm (rua.csv) and right thigh (rt.csv). Each file contains the overall sequence recorded during the experiment. The first column contains a label "qags" indicating the type of recorded data (quaternions, accelerometer, gyroscope data). The next column is the timestamp expressed in milliseconds elapsed from 00:00:0.000 AM (with a 30 milliseconds sampling time). The next four columns are the quaternions (with a resolution of 0.0001). Following them, we have three columns with the accelerations along the x-, y- and z-axis (with a 0.1 mG resolution). The last three columns refer to the angular velocity about the x-, y- and z-axis (with a resolution of 0.01 degrees per second – dps). The last csv file (annotation.csv) contains the data labelling. The first two columns of this file report the current day time in the format hh.mm.ss.000, and in milliseconds elapsed from 00:00:0.000 AM. All the remaining columns are organised as couples where the first element represents the scope of the labelling and the second indicates whether the labelled activity starts or ends. In the annotation file there are four different labelling scopes.

• “BothArms” is used when all instances of each activity are labelled independently of which arm has been employed;
• “RightArm” is used for those activity instances using only the right arm, or in case they belong to the *Walk, Sit Down* or the *Stand Up* activities;
• “LeftArm” is used for those activity instances using only the left arm, or in case they belong to the *Walk, Sit Down* or the *Stand Up* activities;
• “Locomotion” is a scope labelling only the instances of *Walk, Sit Down* and *Stand Up*.

**Table 1:** List and description of the considered activities.

ID	Activities	Category	Description
1	Walk	Transferring/Mobility (Basic)	Moving from seated to standing, getting in and out of bed, and the ability to walk autonomously from one location to another.
2	Sit Down (chair)		
3	Stand Up (chair)		
4	Open Door		
5	Close Door		
6	Pour Water	Eating (Basic)	The ability to feed oneself, though not necessarily the capability to prepare food.
7	Drink Glass		
8	Brush Teeth	Personal hygiene (Basic)	Bathing (e.g., showering, grooming, nail care and oral care).
9	Clean Table	Housework (Instrumental)	Cleaning and maintaining the house.

Finally, the last two columns report a session identification (ID). There are four different sessions characterised by the order in which the activities are performed, the used arm (see also Table 3), and whether the session starts or ends. The videos recorded during the experiments have been used only for labelling purposes, and they are not published.

## 2 Experimental design, materials, and methods

### 2.1 Equipment

In our experiment 6 IMUs (see Table 2 for the related specification), worn by a volunteer as shown in Fig. 1, communicate via Bluetooth with a workstation. When using a Bluetooth-based communication channel, a few sensory data may be lost. In our case, the data drop rate of each sensor has been experimentally evaluated at being below 2%. The IMUs sampling frequency used for these experiments is 33 Hz. IMUs are worn so that the x-axis is pointing downward, and the y-axis is pointing forward, except for the sensor located on the volunteer's back whose y-axis is pointing towards the volunteer's left arm. Furthermore, an RGB camera, i.e., a Logitech HD Pro Webcam C920, is connected to the workstation via USB. The software ALTIMA [2] from ATR-Promotions, is used to record and synchronize the data originating from all the sensors.

**Table 2:** IMU sensor specifications.

Name	TSND 151
CPU	Renesas Technology RX631
Size	40 mm (W) x 50 mm (H) x 14 mm (D)
Weight	About 27 g
Wireless transmission	Bluetooth Ver2.0 + EDR Class 2
Memory storage	2 Gbit
Accelerometer / Gyroscope	InveSense MPU-9250
	Sampling: up to 1000 Hz (1 to 255 ms cycle)
	Accelerometer range: ± 2G / ± 4G / ± 8G / ± 16G
	Gyroscope range: ±250 dps / ±500 dps / ±1000 dps / ±2500 dps

The reference frame of each sensor is set to the frame of the IMU corresponding to the moment when the sensor is connected to the ALTIMA software. In order to have a common reference frame for all the IMUs and among all the experiments, before the connection is activated, we align all the sensors on a table, all of them with the same orientation. This is done manually, and the edge of the table is used to help positioning the sensors all with the same attitude. In this configuration, the IMU-centred reference frames differ only by a translation along the direction of the table's edge.

### 2.2 Environment

**Fig. 2:**
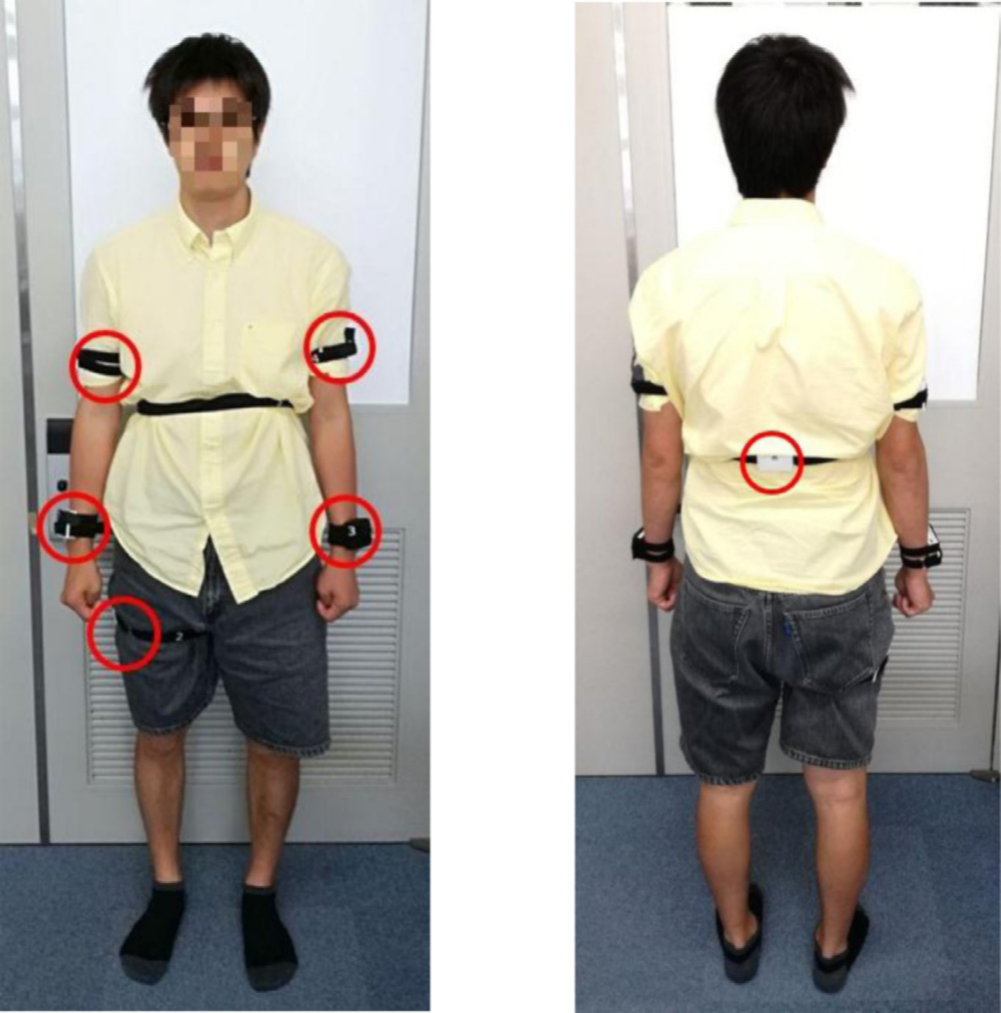
Position of the IMU sensors on the volunteer's body.

In Fig. 2 a map of the environment where the experiments took place is shown. The environment consists of a room with a table, two chairs, a washbasin and two doors. Two dinnerware sets, each one composed of a cup and a half litre bottle, have been positioned on the table close to the chairs. A personal hygiene set has been located close to the washbasin, and it includes a personal toothbrush. Each location where the activities are performed are identified by a letter. Overall, there are three main locations, namely A, B, and C. The distance between them is approximately 4 meters between A and C, 3 meters between B and C, and 1 meter between A and B. Fig. 2 shows the position of the workstation used for data collection as a yellow rectangle, and the RGB camera as a yellow triangle.

**Fig. 3:**
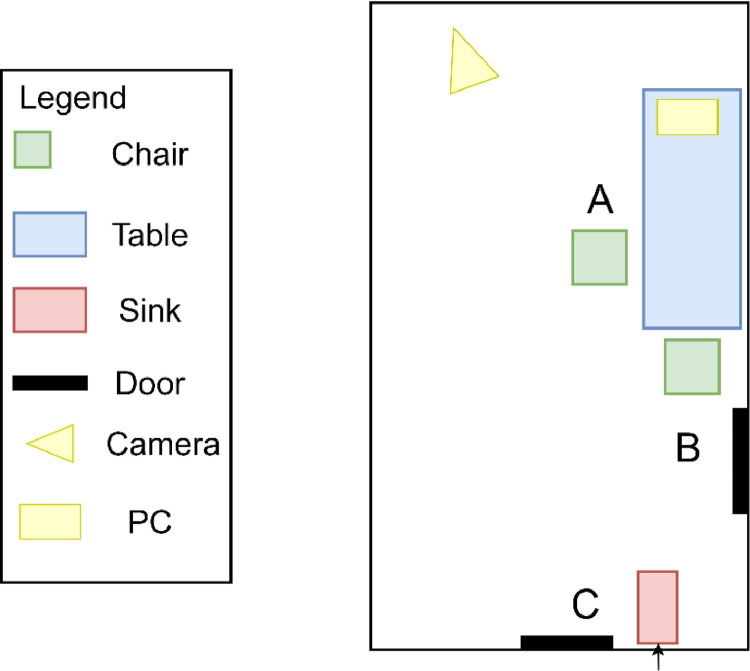
The map of the environment where the experiments took place.

## 3 Experimental Setup

In order to record ADL-related data as shown in Table 1, we have carefully designed two distinct activity sequences, as shown in Table 3. The sequences have been designed with the goal of maintaining a balanced number of repetitions for each activity while recording sequences that are long enough to be considered realistic. Since all the activities, except for *Walk, Sit Down* and *Stand Up*, involve the usage of one hand only, we asked all the volunteers to carry out each sequence twice: using only the right hand (sessions S1 and S3 in Table 3), and the left hand (sessions S2 and S4 in Table 3), respectively. This led to four experimental scenarios, which have been carried out sequentially. The sequence descriptions in Table 3 contain the expected volunteer's initial and final poses, and the location where each activity should be performed. The locations are referred to with the letters A, B and C introduced above, and can help the experimenter to keep track whether the experiment's execution progresses as expected. Except for the arm used to perform the activities, no indication about task execution is given to the volunteers. Therefore, the execution time of each activity can vary significantly. This is particularly evident for the *Walk* activity due to the different distances among the three predefined locations.

**Table 3:** Order in which the activities are performed for each sequence.

Session ID	S1 (right hand)		S3 (right hand)	
	S2 (left hand)		S4 (left hand)	
**Initial Positions**	Sit	A	Sit	A
**Activity Sequence**	**Activity**	**Location**	**Activity**	**Location**
1	Drink Glass	A	Stand Up	A
2	Pour Water	A	Clean Table	A
3	Stand Up	A	Sit Down	A
4	Walk	A→B	Pour Water	A
5	Open Door	B	Stand Up	A
6	Clean Table	B	Drink Glass	A
7	Walk	B→A	Walk	A→C
8	Sit Down	A	Drink Glass	C
9	Pour Water	A	Open Door	C
10	Drink Glass	A	Close Door	C
11	Stand Up	A	Pour Water	C
12	Walk	A→C	Walk	C→B
13	Open Door	C	Pour Water	B
14	Brush Teeth	C	Open Door	B
15	Walk	C→B	Pour Water	B
16	Clean Table	B	Walk	B→C
17	Sit Down	B	Open Door	C
18	Drink Glass	B	Drink Glass	C
19	Pour Water	B	Brush Teeth	C
20	Stand Up	B	Pour Water	C
21	Walk	B→C	Close Door	C
22	Close Door	C	Drink Glass	C
23	Brush Teeth	C	Walk	C→B
24	Walk	C→B	Drink Glass	B
25	Clean Table	B	Close Door	B
26	Close Door	B	Clean Table	B
28	Walk	B→A	Pour Water	A
29	Sit Down	A	Sit Down	A
30	Stand Up	A	Drink Glass	A
31	Pour Water	A	Clean Table	A
32	Clean Table	A	Pour Water	A
33	Drink Glass	A	Stand Up	A
34	Walk	A→B	Walk	A→C
35	Sit Down	B	Open Door	C
36	Clean Table	B	Pour Water	C
37	Stand Up	B	Brush Teeth	C
38	Walk	B→C	Drink Glass	C
39	Brush Teeth	C	Walk	C→B
40	Walk	C→B	Clean Table	B
41	Close Door	B	Open Door	B
42	Walk	B -> C	Walk	B→A
43	Brush Teeth	C	Drink Glass	A
44	Open Door	C	Sit Down	A
45	Brush Teeth	C	Stand Up	A
46	Close Door	C	Walk	A→C
47			Close Door	C
**Final position**	Standing	C	Standing	C

The correct execution order of each sequence is guaranteed by the experimenter, who communicates the next activity to perform to the volunteer. However, a few errors in the execution of the sequence have occurred during the experiments. These errors do not alter the validity of the dataset as they consist only in a deviation from the plan detailed in Table 3, and are properly labelled. For this reason, the order of execution of the activities presented in the labelling files can slightly diverge with respect to the sequences presented in Table 3.

Before sensors are worn by the volunteers, the IMUs are positioned on a table in clear sight of the camera. They are rotated, one at a time, by 90 degrees about their z-axis in one direction, and then in the opposite direction back to the initial reference pose. This is done to facilitate the removal of possible offsets for the synchronization of IMUs and camera data.

After this setup, and before starting the experiment, a calibration motion is executed by the volunteers to check that all the sensors are positioned correctly on their body. The calibration motion consists in moving one body part at a time. First, the right leg is lifted pulling the knee close to the chest, followed by the left leg. Then, the right arm is lifted laterally, and the elbow is bent so that the lower arm forms a 90-degree angle with the upper arm. The elbow is straightened back, and the arm is put at rest along the body. The same motion is done with the left arm. The calibration phase is repeated at the end of the sequence as well.

The execution of each scenario lasts approximately for 4 minutes, while the sensors setup and the experiment explanation take 10 minutes. Therefore, the overall length of the experiment is about half an hour for each volunteer.

### 3.1 Volunteers

The dataset has been collected by 10 healthy volunteers whose relevant information is summarized in Table 4. All the volunteers are right-handed and aged between 22 and 28. Data are collected from healthy people, i.e., without any physical condition that could affect the execution of certain tasks, and young subjects have been preferred. Furthermore, the selection of the volunteers tried to balance a gender-related representation in the data (3 females and 7 males), and includes subject with different heights (from 156 cm to 187 cm) and weights (from 45 kg to 77 kg).

**Table 4:** Participants Information.

Person ID	Sex	Age	Dominant Hand	Height (cm)	Weight (kg)	Nationality
P01	M	22	Right	172	62	Japan
P02	M	25	Right	187	77	Japan
P03	M	25	Right	169	70	Japan
P04	F	24	Right	156	55	Japan
P05	F	23	Right	161	50	Japan
P06	F	24	Right	163	45	Japan
P07	M	24	Right	166	55	Japan
P08	M	23	Right	173	68	Japan
P09	M	27	Right	173	65	Japan
P10	M	28	Right	178	70	Italy

### 3.2 Synchronization and labelling

IMU- and camera-related data must be synchronized in order to use video streams to label IMU data. Both the synchronization and labelling processes are carried out using the SyncPlay [3] software from ATR-Promotions. The ALTIMA software, used for data recording, already synchronizes the camera and the IMUs sampling time. However, sometimes an offset between the two data streams is present. In SyncPlay, it is possible to visualize IMU data along with the video and to modify the initial time of the video to synchronize it with the IMU data stream. Therefore, the rotation of the IMUs while on the table, as described above, is used to remove any offset.

The labelling process requires defining the start and end events for each activity. In the following paragraphs, we report the policies we used for the labelling procedure.

• *Walk* starts when the first leg moves to perform the first step, and it ends when both legs have stopped. These bounds do not include little steps performed to change the body orientation, or single steps aimed at getting closer to doors or tables to perform another activity.
• *Sit Down* starts when the person alters their vertical body position in order to sit. The actual motion may vary from person to person, and more in general the sitting motion depends on the chair type and on the location where the chair is positioned with respect to the person. The *Sit Down* motion ends when the person sitting posture is relaxed, showing that the motion has ended.
• *Stand Up* starts when the person initiates to move the body weight from the chair to the feet. This is clearly visible when the person moves forward the torso. This motion ends when the person reaches the normal standing height, after stretching the legs completely.
• *Open Door* starts when the hand of the person touches the door handle and ends when the door is completely open.
• *Close Door* starts when the hand of the person touches the handle or any other part of the door and ends when the door is completely closed or when the hand of the person releases the door.
• *Pour Water* starts when the person touches the bottle and ends when their hand releases it.
• *Drink Glass* starts when the person touches the glass for the first time and ends when the hand is not in contact anymore with it.
• *Brush teeth* starts when the toothbrush enters in contact with the teeth for the first time and ends when the toothbrush leaves the mouth.
• *Clean Table* starts when the hand enters in contact with the table for the first time and ends when the contact regime ends.

The labelling process has been performed manually by one experimenter visualising the experiment videos with SyncPlay. Annotations are made when activities start or end. The time format in SyncPlay is hh.mm.ss.000, while the ALTIMA software saves timestamps in millisecond elapsed from 00:00:00.000 AM. Therefore, the time stamp has been converted as follows:

(1) $$msFrom0AM=ss.000*10^{3}+mm*6*10^{4}+hh*3.6*10^{6}$$

As described above, the labelling process is performed considering four scopes, namely *Both Arms, Right Arm, Left Arm* and *Locomotion*. When an activity instance does not fit one of those scopes, it receives a *null* label for that specific scope. For example, brushing the teeth with the right hand will result in a null label in both *Left Arm* and *Locomotion*.

For each experiment, the time required to perform the labelling amounts approximately to 2 hours.

Together with the dataset, we provide a MATLAB script named *TimeStampExtraction.m* that extracts from the annotation and data files, for each volunteer and for each sensor, the time stamp associated with the start and end time of each ADL.

## Ethics statement

The study was performed in accordance with the Helsinki Declaration and approved by the institutional Ethics Committee of Keio University (31-79 2019). All the subjects participated voluntarily and gave their written, informed consent.

## Declaration of Competing Interest

The authors declare that they have no known competing financial interests or personal relationships which have, or could be perceived to have, influenced the work reported in this article.

## Appendix Supplementary materials

Image, application 1

## Footnotes

1 https://data.mendeley.com/datasets/wjpbtgdyzm/1
